# Comparison of the therapeutic effect of platelet-rich plasma and injectable platelet-rich fibrin on testicular torsion/detorsion injury in rats

**DOI:** 10.1038/s41598-024-67704-4

**Published:** 2024-08-05

**Authors:** Eslam F. M. Eisa, Shimaa A. M. Ezzeldein, Haiam A. Mohammed, Asmaa A. Abdallah, Wael A. M. Ghonimi, Mustafa Abd El Raouf

**Affiliations:** 1 Department of Surgery, Anesthesiology and Radiology, Faculty of Veterinary Medicine, Zagazig University, Zagazig, 44519 Egypt; 2 Department of Physiology, Faculty of Veterinary Medicine, Zagazig University, Zagazig, 44519 Egypt; 3 Department of Theriogenology, Faculty of Veterinary Medicine, Zagazig University, Zagazig, 44519 Egypt; 4 Department of Histology and Cytology, Faculty of Veterinary Medicine, Zagazig University, Zagazig, 44519 Egypt

**Keywords:** Testicular torsion, Torsion/detorsion, Semen, Oxidative stress, PRP, i-PRF, Medical research, Urology

## Abstract

Testicular torsion is a common disorder in males and results in blockage of testicular circulation with subsequent damage of testicular germ cells. The current work aimed to compare the therapeutic effect of platelet-rich plasma (PRP) and injectable platelet-rich fibrin (i-PRF) on torsion/detorsion (T/D) injury in rats. Forty mature male Wister rats were arranged into 4 groups; (1) Control, (2) T/D, (3) T/D + PRP, and (4) T/D+ i-PRF. The right testis was twisting 1080° clockwise for 3 h in groups 2, 3 and 4, then 10 μl of PRP or i-PRF was injected intra-testicular 3 h after detorsion in groups 3 and 4, respectively. After 30 days postoperatively, the semen quality and hormonal assay were improved in PRP and i-PRF-treated groups with superiority of i-PRF (*P* < 0.001). High significance of Catalase, Glutathione Peroxidase (GPx), Superoxide Dismutase, Interleukin-1β (IL-1β), Caspase-3 and Tumor necrosis factor-α (TNF-α) was reported in treated rats with PRP and i-PRF (*P* < 0.001) with superiority to i-PRF-treated rats (*P* < 0.001). Testicular histoarchitectures were improved in PRP and i-PRF-treated rats with superiority of i-PRF-treated rats. It was concluded that PRP and i-PRF have regenerative efficacy on testicular damage after induced T/D injury with a superior efficacy of i-PRF.

## Introduction

In the field of urology, the most commonly recorded disorder in males is testicular torsion. This disorder leads to blood flow impairment and testicular germ cells damage due to rotation of the testis around its spermatic cord^1^. It is an emergent disorder and more commonly observed among children and adolescents of 1–25 years old for about 4.5 in 100,000 male^2^. It causes reduction in fertility rate of the patients as spermatogenesis decreased in 50–95% of patients with loss of ipsilateral testis^3^. The prognosis depends mainly on the onset of testicular torsion and its degree that affect the sperm survival and activity^4^. Therefore, it is of great importance to diagnose this case in early stage and determine the proper surgical interference for correction in order to prevent testicular damage with subsequent infertility^5^.

Testicular torsion/detorsion (T/D) results in testicular biochemical and morphological alterations^6^. Reperfusion after testicular ischemia causes increased reactive oxygen species (ROS) production and inflammatory cytokines activation^7,8^, which cause oxidative stress of the cells and resulting in cellular dysfunction and apoptosis^9^. Moreover, reperfusion is linked also to the overproduction of Interleukin-1β (IL-1β) and Tumor necrosis factor-α (TNF-α) in addition to chemokines and cell adhesion molecules resulting in neutrophil and macrophage recruitment^10,11^.

Platelet rich plasma (PRP) is considered the first generation of plasma concentrates which contains multitude of platelets folded than its number in the blood^12^. The PRP has several growth factors that promote healing process through enhancing angiogenesis, and proliferation and differentiation of cells^13,14^. So, it is used in different fields of regenerative medicine such as plastic, dental and orthopedic surgeries^15,16^. Some limitations were reported about the use of PRP due to addition of anticoagulant during preparation and the rapid growth factors release after its activation^17^.

Platelet-rich fibrin (PRF) is another form of platelet concentrates which contain leukocytes and platelets distributed in a fibrin matrix produced without any additives^18^. Its use promotes the soft and hard tissues regeneration^19^. During the healing process of wounds, cytokines are utilized and eliminated almost instantly. The growth factors release from the fibrin clot is slow and prolonged in comparison to PRP^20^. More recently, liquid form of PRF known as injectable-PRF (i-PRF) has been obtained with lowering the centrifugation speed and time. Injectable-PRF contains higher levels of platelets and leukocytes and provides a controlled system for growth factors release during the process of healing^21^. This form of i-PRF remained liquid for 15–20 min. The time of clotting might be improved by using chemically modified PET tubes for over 20 minute^22^. It has been reported that i-PRF has greater ability for induction of collagen synthesis and fibroblast migration and differentiation in comparison to PRP^23^. It is considered as a new alternative to PRP in various medical practices and to traditionally produced PRF^24–26^.

PRP has been evaluated for its regenerative ability on testicular T/D injury^27–29^. There was no available data about the effect of i-PRF on testicular degeneration after testicular torsion. Therefore, the current work aimed to compare the therapeutic effect of PRP and i-PRF on T/D injury in rats.

## Results

### Effects of T/D on semen evaluation

The quality of sperms collected from the cauda epididymis of the ratś testes was different in all groups as listed in Table 1. Semen samples from the T/D and PRP groups had degenerative sperms, while those of control and i-PRF groups had nearly equal sperm counts (118.3 ± 13.8 and 116.6 ± 21, respectively). Regarding sperm motility, there was no motility in the T/D and PRP groups. Interestingly, the sperm motility in the i-PRF group was 50 ± 11.54% in comparison to the control group which had 88.3 ± 4.4% of sperm motility. Concerning abnormalities of the sperms, all sperms in the T/D and PRP groups had abnormalities, while control and i-PRF groups had lower abnormalities (10.01 ± 0.26% and 25.6 ± 3.7%, respectively).

**Table 1:** Effects of T/D on semen evaluation.

	Sperm count (mL× 125×10^4 )	Sperm motility (%)	Sperm abnormalities (%)
Control	118.3±13.8^a^	88.3±4.4^a^	10.01±0.26^a^
T/D	Degenerated sperms	0^c^	100^c^
PRP	Degenerated sperms	0^c^	100^c^
i-PRF	116.6±21^a^	50±11.54^b^	25.6±3.7^b^

Different superscript letters indicates significance (*P* < 0.05). The data are presented as means ± SD.

### Effects of T/D on reproductive hormones’ levels

**Figure 1:**
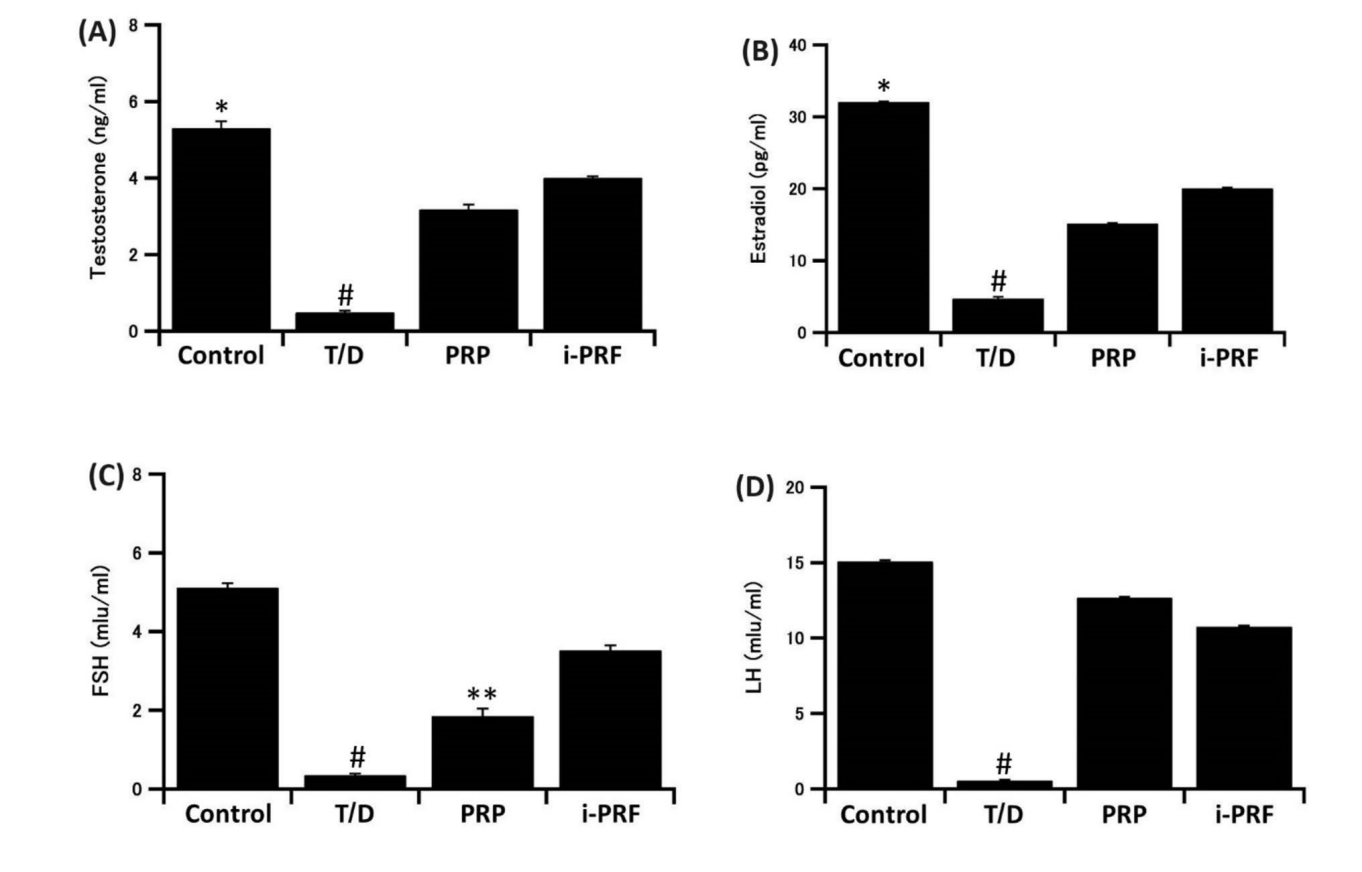
Effects of T/D on serum levels of (**A**) Testosterone, (**B**) Estradiol, (**C**) FSH, and (**D**) LH. #: significantly lower than all other groups; *: significantly higher than the other two groups; **: significantly lower than the other two groups. The data are presented as means ± SD.

Levels of testosterone, estradiol, luteinizing hormone (LH) and follicular stimulating hormone (FSH) in the serum were decreased significantly in the T/D group as compared to the control group (*P* < 0.001). Those levels were reestablished significantly (*P* < 0.001) after treatment with PRP and i-PRF. Interestingly, in the treated groups, the highest levels of testosterone, estradiol, and FSH were recorded in the i-PRF-treated group, while the PRP-treated group recorded the highest level of LH (Fig. 1).

### Effects of T/D on antioxidants and lipid peroxidation levels in testicular tissue

**Figure 2:**
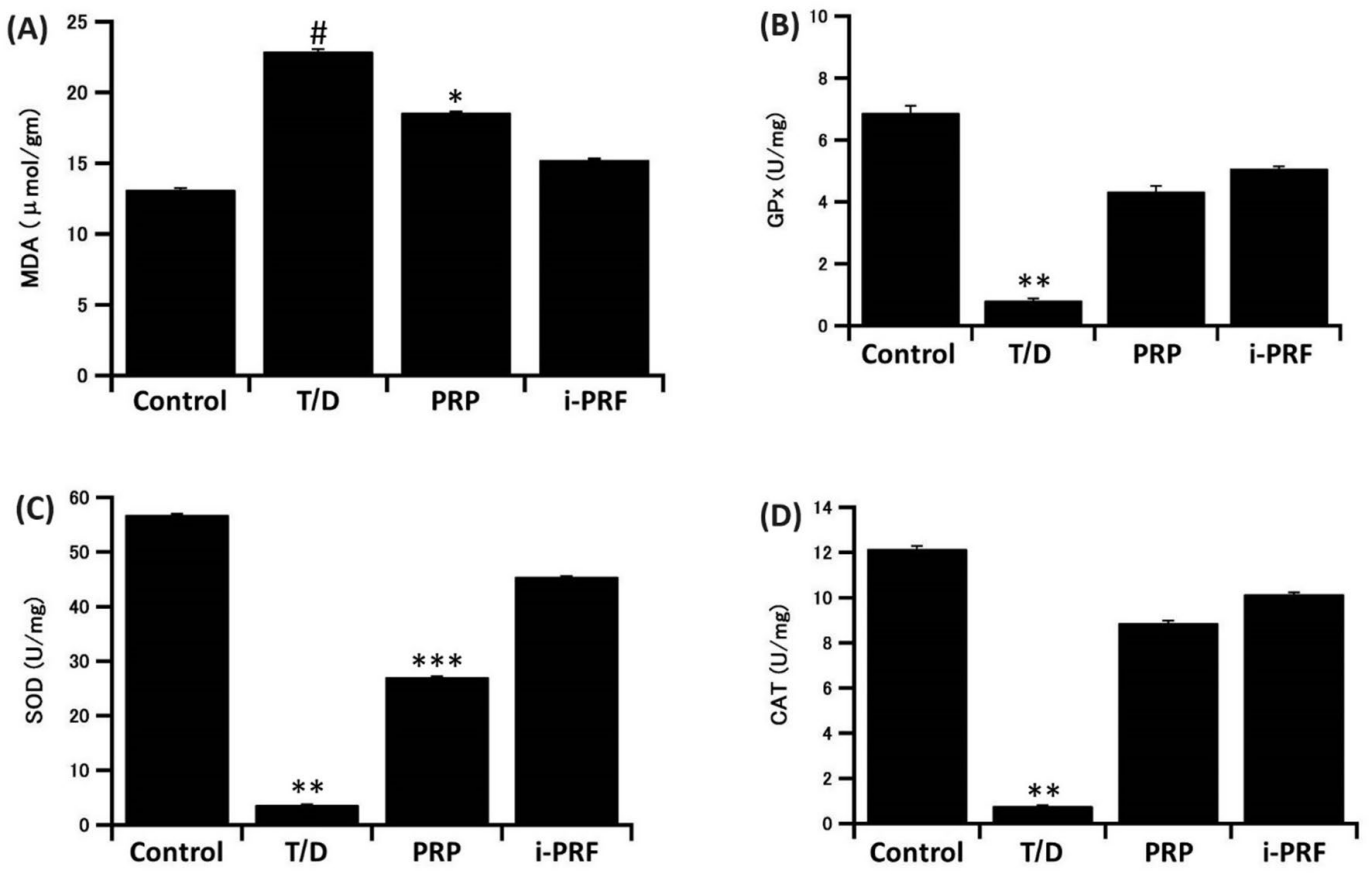
Effects of T/D on antioxidants and lipid peroxidation levels of (**A**) MDA, (**B**) GPx, (**C**) SOD, and (**D**) CAT in testicular tissue. #: significantly higher than all other groups; *: significantly higher than the other two groups; **: significantly lower than all other groups; ***: significantly lower than the other two groups. The data are presented as means ± SD.

A significant rise in testicular MDA level (*P* < 0.001) simultaneously with significant depression in the testicular CAT, SOD and GPx levels (*P* < 0.001) were observed in T/D group as compared to the control one. All those levels were significantly reversed in PRP and i-PRF groups as compared to the T/D group. Notably, the highest levels of CAT, GPx and SOD were recorded in i-PRF treated group which recorded also the lowest level of MDA in comparison to PRP-treated group (Fig. 2).

### Effects of T/D on the gene expression of Caspase-3, TNF-α and IL-1β in testicular tissue

**Figure 3:**
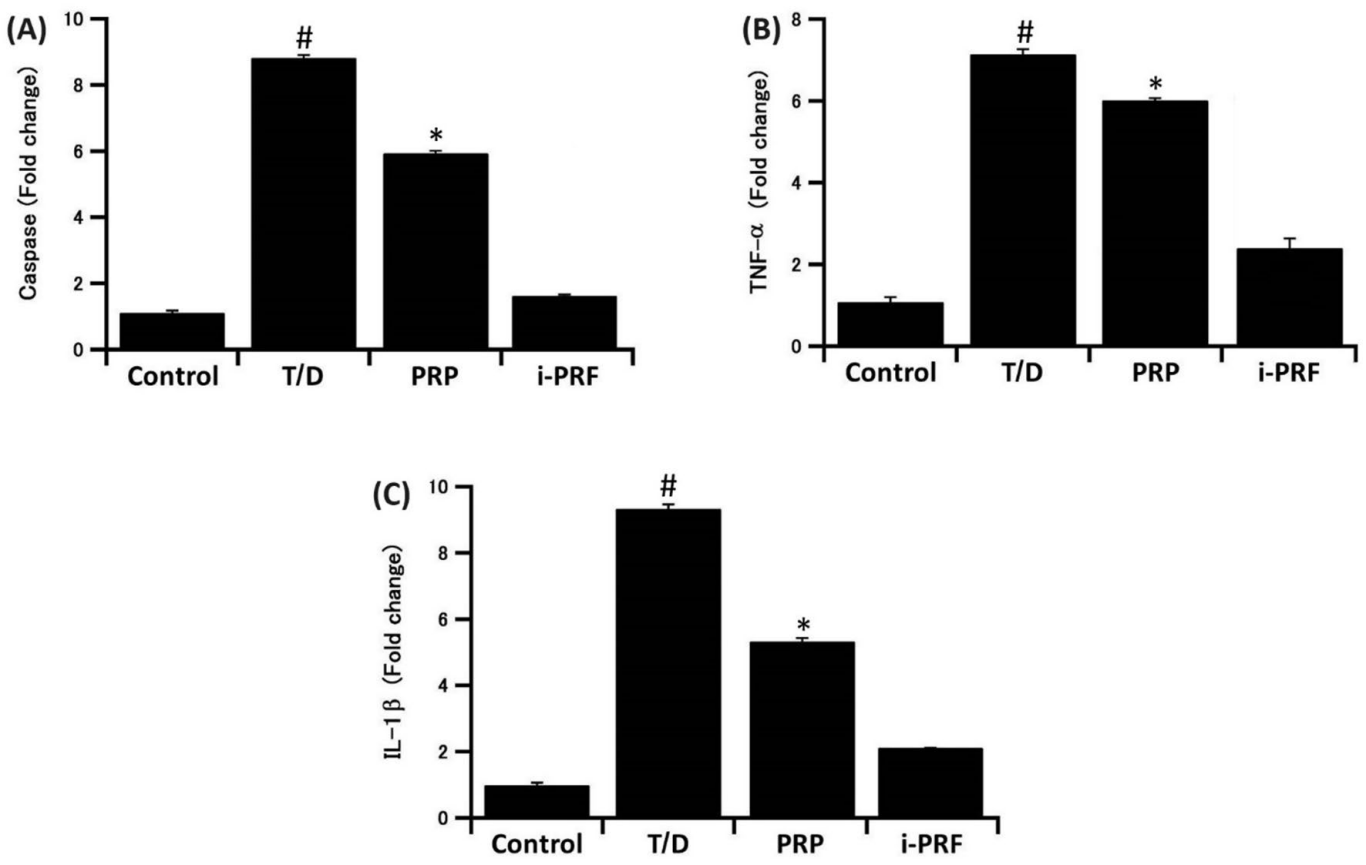
Effects of T/D on gene expression of Caspase-3 (**A**), TNF-α (**B**), and IL-1β (**C**) in testicular tissue. #: significantly higher than all other groups; *: significantly higher than the other two groups. The data are presented as means ± SD.

Caspase-3, IL-1β and TNF-α expressed significant (*P* < 0.001) increase in T/D group. In contrast, the expression of the three genes was significantly (*P* < 0.001) downregulated in PRP and i-PRF- treated groups as compared to the T/D group. Interestingly, Caspase-3, IL-1β and TNF-α expressed lower value in i-PRF treated group than PRP treated group (Fig. 3).

### Effects of T/D on the histological architecture of the testes

**Figure 4:**
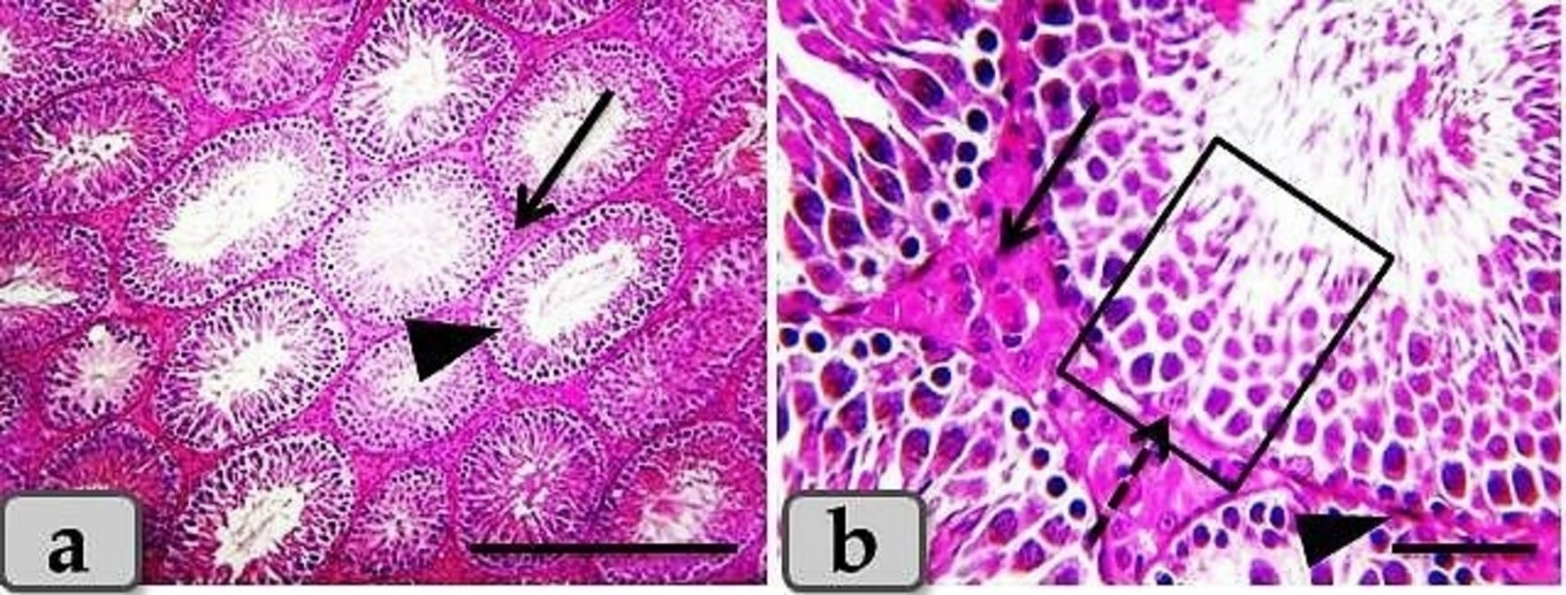
(**a**, **b**) Photomicrographs of the adult male rats testes of the control group; a) showing normal, intact testicular parenchyma of tubular part (oval or rounded seminiferous tubules) (arrow head) and intertubular part (A considerable amount of highly vascularized interstitial connective tissue housing leydig and myoid cells) (arrow). (**b**) Showing normal, intact seminiferous tubules lining stratified seminiferous epithelium; pyramidal sertoli cells (dashed arrow) surrounded with several rows of normal, organized, proliferating highly divided spermatogenic cells (inside square) rested on a thin basal lamina, also, showing intact ovoid or polygonal leydig cells (arrow) and flat myoid cells (arrow head) in the intertubular part. Stain: (**a**, **b**) H&E. Scale bars: a = 300 µm, b = 40 µm.

The control group revealed normal, intact testicular parenchyma that was mainly consisted of two parts; tubular part (numerous oval or rounded seminiferous tubules) and intertubular part (A considerable amount of highly vascularized interstitial connective tissue). With higher magnification, the seminiferous tubules were lined with intact stratified seminiferous epithelium that was composed of non-divided fewer pyramidal sertoli cells surrounded with several rows of normal, organized, proliferating highly divided spermatogenic cells rested on a thin basal lamina and represented by spermatogonia, spermatocyte I, spermatocyte II, spermatids and sperms. Highly vascularized intertubular connective tissue appeared housing two types of cells; ovoid or polygonal leydig cells with spherical nuclei and flat myoid cells with flat nuclei (Fig. 4).

**Figure 5:**
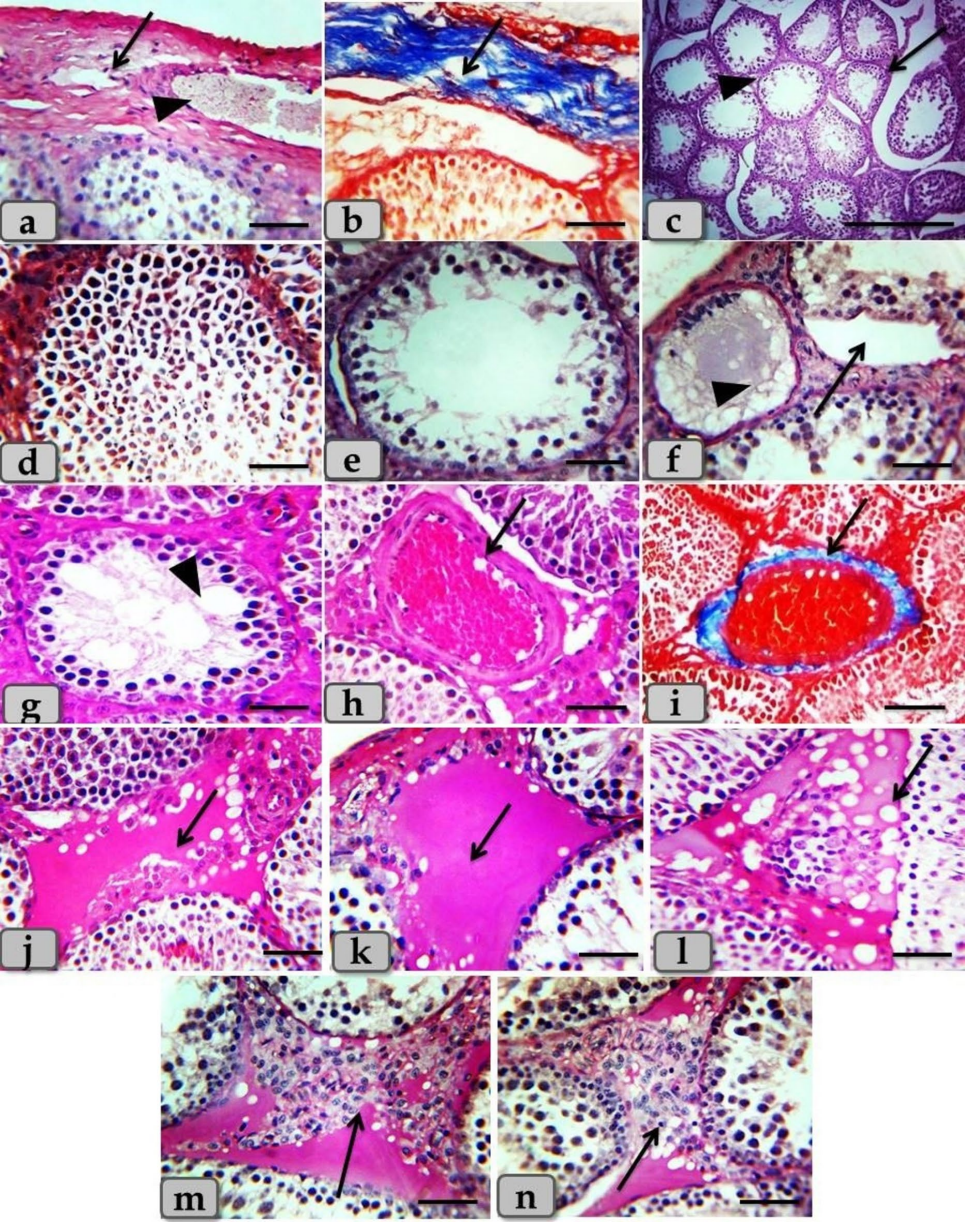
(**a**–**n**) Photomicrographs of the mature male ratś testes of the T/D group; (**a**, **b**) Showing thickening and fibrosis of the testicular capsule (arrow) housing dilated blood vessels (arrow head). (**c**) Showing atrophy of the seminiferous tubules (arrow), thickening of its basal lamina (arrow head) with increasing the intertubular spaces. (**d**) Showing severe necrosis and degeneration of the seminiferous tubules lining epithelium with a loss of their normal organization, in addition, distribution of numerous spermatogenic cells with a pyknotic nucleus in the tubular lumen. (**e**) Showing loss of several types of spermatogenic cells especially spermatocytes II, spermatids and sperms and only two types of cells were present; spermatogonia rest on thick basal lamina and spermatocytes I with pyknotic nucleus, also, the lumen of the seminiferous tubules become more wide and devoid from any sperms. (**f**) Showing severe damage of the seminiferous tubuleś basal lamina with sloughing of its lining epithelium into the lumen (arrow), also some seminiferous tubules were completely devoid from any lining cells and filled with fluid that reacted negatively with PAS stain (arrow head). (**g**) Showing numerous vacuoles of variable shape and size distributing in between the seminiferous epithelium (arrow head). (**h**, **i**) Showing severe intertubular blood vessels dilatation, congestion, with thickening, and fibrosis of its wall (arrow). (**j**) Showing increased the intertubular fluid (arrow). (**k**) Showing PAS positive reactivity of the intertubular fluid (arrow). (**l**) Showing severe hydropic or vacuolar degeneration represented in the distribution of numerous vacuoles of variable shape and size in the intertubular part (arrow). (**m**, **n**) Showing severe leydig cells hyperplasia (arrow).. Stain: (**g**, **h**, **j**, **l**) H&E, (**b**, **d**, **i**) Blue Masson’s Trichrome, (**a**, **c**, **e**, **f**, **k**, **m**, **n**) PAS. Scale bars: All = 40 µm, except c = 300 µm.

Meanwhile, the testicular tissues after T/D revealed thickening and fibrosis of the testicular capsule; tunica albuginea, atrophy of the seminiferous tubules with thickening of its basal lamina, severe necrosis and degeneration of the seminiferous tubules lining epithelium with loss of their normal organization, in addition, distribution of numerous spermatogenic cells with pyknotic nucleus in the tubular lumen. Some examined sections clarified loss of several types of spermatogenic cells especially spermatocytes II, spermatids and sperms and only two types of cells were observed; spermatogonia rest on thick basal lamina and spermatocytes I with pyknotic nucleus, also, the lumen of the seminiferous tubules became more wide and devoid from any sperms. In addition, some sections clarified severe damage to the seminiferous tubuleś basal lamina with sloughing of its lining epithelium into the lumen. Also some seminiferous tubules were completely devoid from any lining cells and filled with fluid that reacted negatively with PAS stain. Furthermore, numerous vacuoles with a different shape and size distributed in between the seminiferous epithelium (Fig. 5). Moreover, the intertubular parts were characterized by several pathological changes resembling sever dilatation and congestion of the intertubular blood vessels, a severe thickening of its wall with proliferations of fibrous connective tissue and fibrosis, a severe increase of the intertubular fluid that reacted positively with PAS stain, with severe hydropic or vacuolar degeneration represented in the distribution of numerous vacuoles with different shape and size in the intertubular part, accompanied with severe leydig cells hyperplasia (Fig. 5).

**Figure 6:**
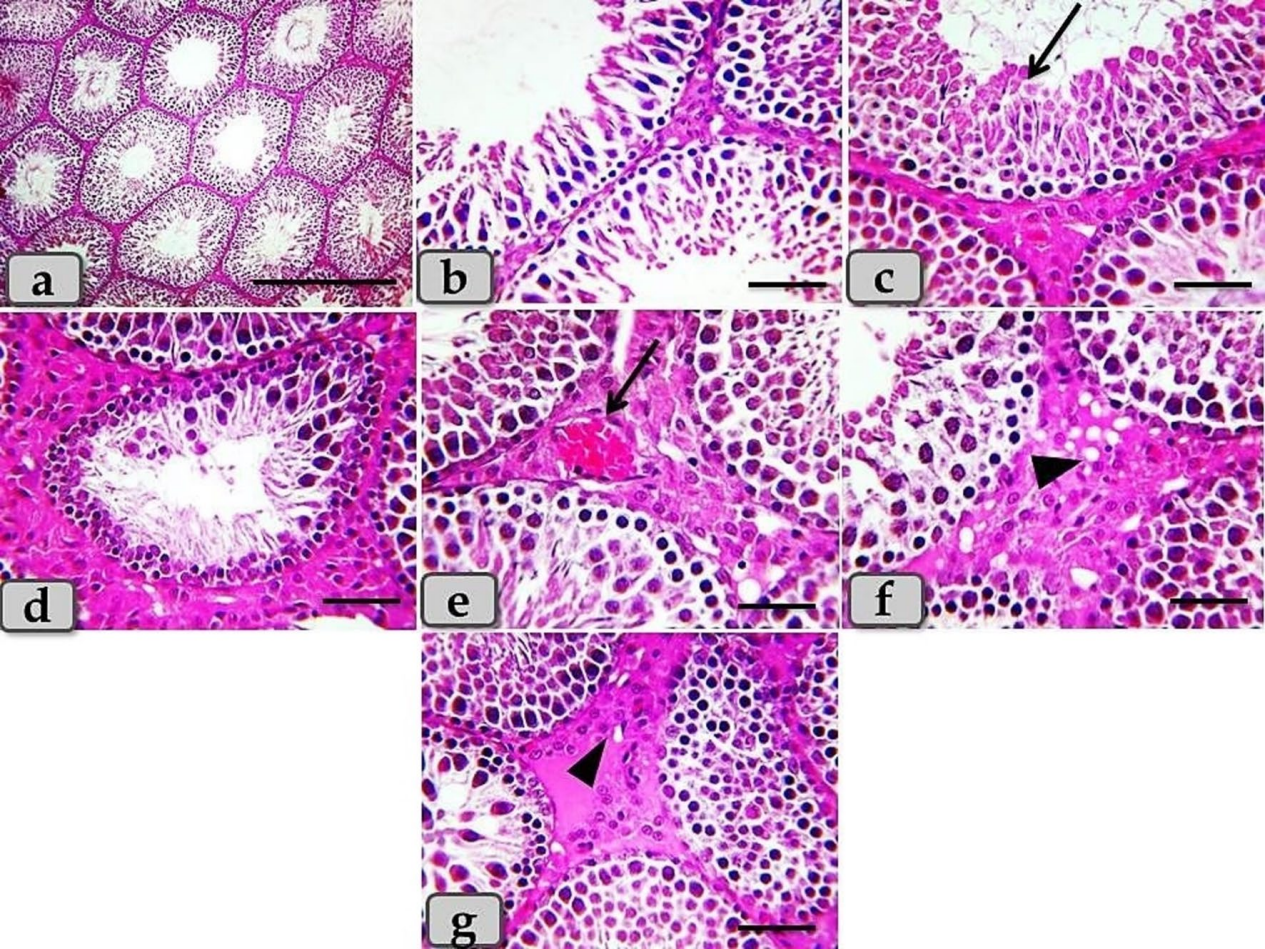
(**a**–**g**) Photomicrographs of the mature male ratś testes of the PRP treated group. (**a**) Showing intact testicular parenchyma of normal tubular part and intertubular part. (**b**) Showing intact seminiferous tubules lining stratified seminiferous epithelium with normal, organized, spermatogenic cells rested on a thin basal lamina, but with moderate pathological changes as necrosis of some spermatogenic cells especially for spermatid and sperms. (**c**) Showing moderate coagulative necrosis of spermatids and sperms (arrow). (**d**) Showing intact seminiferous tubules but with moderate necrosis of spermatogenic cells with loss of some types of cells, also spermatogonia & primary spermatocytes with pyknotic nucleus were clarified. (**e**) Showing moderate congestion of the intertubular blood vessels (arrow). (**f**) Showing mild to moderate vacuolar degeneration in the intertubular part (arrow head). (**g**) Showing moderate leydig cells hyperplasia in the intertubular part (arrow head). Stain: All) H&E. Scale bars: All = 40 µm, except a = 300 µm.

Regarding the testicular tissues of the PRP treated group, PRP clarified a moderate therapeutic effect after T/D. The examined sections clarified intact testicular parenchyma of normal tubular part and intertubular part. And also, with magnification, intact seminiferous tubules lined with stratified seminiferous epithelium were observed, with normal, organized, spermatogenic cells rested on a thin basal lamina, but with moderate pathological changes as degeneration of some spermatogenic cells especially for spermatid and sperms (Fig. 6). In addition, moderate coagulative necrosis of spermatids and sperms were noticed, with moderate necrosis of spermatogenic cells accompanied with loss of some types of cells. Also, spermatogonia & primary spermatocytes with pyknotic nucleus were clarified. Furthermore, moderate congestion of the intertubular blood vessels, mild to moderate vacuolar degeneration in the intertubular part, and moderate leydig cells hyperplasia were demonstrated (Fig. 6).

**Figure 7:**
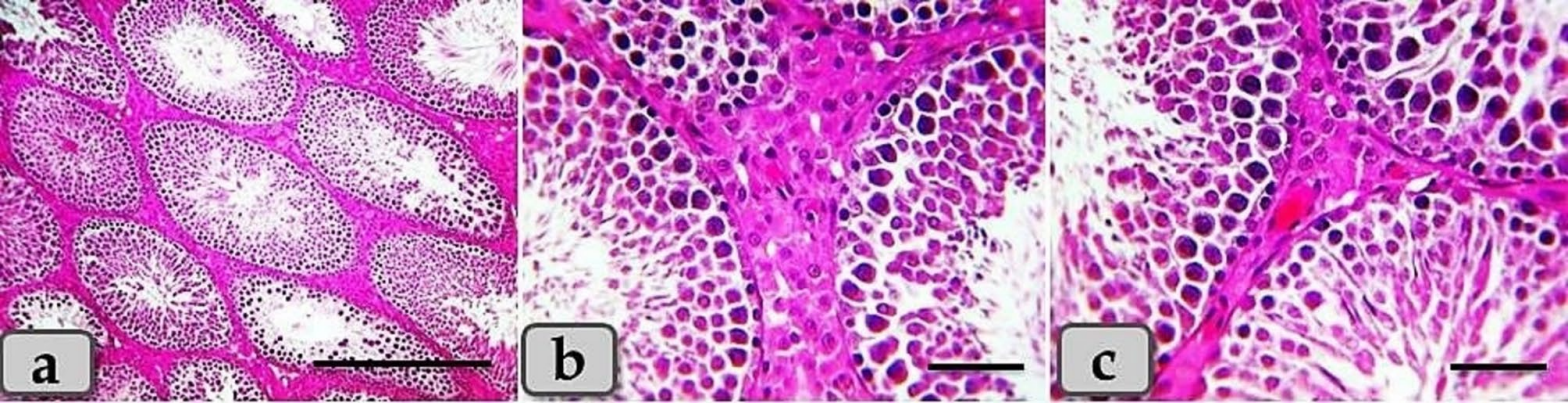
(**a**–**c**) Photomicrographs of the mature male ratś testes of the i-PRF treated group; (**a**) Showing semi normal, intact tubules and intertubular parts that appeared looks like normal resembling the normal control group with very slightly epithelial degeneration in individual tubules. (**b**, **c**) Showing normal, organized stratified seminiferous epithelium without any pathological changes. Stain: All) H&E. Scale bars: All = 40 µm, except a = 300 µm.

Meanwhile, the testicular tissues of the i-PRF-treated group clarified the preferable and effective therapeutic interference after T/D. This group showed normal, intact tubules and intertubular parts that appeared looks like normal resembling the normal control group, with normal, organized stratified seminiferous epithelium in almost of the examined sections and only very slightly epithelial degeneration were observed in some individual tubules (Fig. 7).

## Discussion

Testicular damage caused by testicular torsion has been experimentally studied to illustrate the degenerative changes of the testis and how to manage and counteract theses alterations using different modalities with different materials^27,30^. It was reported that PRP has protective role following low and high degrees of testicular torsion at 720°^29^ and 1080°^27^, respectively. This current work is the first to investigate the regenerative ability of i-PRF after a high degree of testicular torsion at 1080° for 3 h in comparison to PRP.

Infertility is the common outcome of testicular torsion which represents a common medical emergency. It has been reported that the testicular damage after testicular torsion damage resembles ischemia/reperfusion (I/R) injury^31^. Injury accompanying I/R in rat testis leads to formation of ROS, release of IL-1β and TNF-α, stimulation of pro-inflammatory cytokines, activation of nitric oxide synthase, germ cell apoptosis, testicular atrophy, and reduced spermatogenesis^7,32^.

It was reported that testicular torsion leads to necrosis of the germ cell, suppress spermatogenesis, and decreased testosterone serum levels, with consequent infertility^33^. In the current work, the semen quality was significantly reduced after T/D. On the other hand, it was improved in the i-PRF-treated group.

Our data revealed a significant increase in testicular MDA levels and a significant decrease in SOD and GPx levels in T/D group. These findings were obtained in previous studies^11,28,34,35^. In this regard, previous research has demonstrated that testicular T/D reduces SOD activity^36^.

Testicular T/D-induced damage is complicated by oxidative stress. T/D results in a surge of mitochondrial ROS production leading to consumption of natural antioxidants causing oxidative stress. The ROS that have been released have a negative impact on the body causing lipid, protein, and DNA oxidative damage^37^. I/R of testis induces severe peroxidation of the cell membrane which is indicated by the elevated level of MDA. In addition, Ischemia causes oxidative stress and germ cell apoptosis due to a decrease in oxygen availability, cellular energy depletion, and toxic metabolites accumulation with subsequent disruption of cell structure and function^38^.

The PRP-treated group expressed higher levels of SOD than the T/D group. This result agrees with the previous study^28^ where they suggested that PRP reduced testicular injury by lowering ROS levels. Interestingly, the result of the i-PRF treated group in our study still the most effective in restoring the antioxidant status after T/D.

Testosterone, LH, FSH and estradiol serum levels were significantly decreased in the T/D group. They were found to be promoted in treated groups, however; they were still lower than that of the control group. Similar findings were recorded previously^28,35^. It was reported that reduced levels of reproductive hormones were linked to an increase in oxidative stress indicators. Low serum testosterone level represents oxidative stress which is generated in testis caused by I/R injury^39^. Testosterone seems to be responsible for the optimum function of the blood-testis barrier. Failure of spermatogenesis and infertility is the result of disturbance of any process depends on testosterone^40^. Sex hormones and sex hormone-binding hormone have been shown to be important in spermatogenesis and maturation of sperm^41^. In males, LH stimulates the formation of testosterone by Leydig cells subsequently control spermatogonial cell development and Sertoli cells spermatogenesis in conjunction with FSH^42^.

Our data showed that PRP improved serum testosterone level compared to T/D group that was in agreement with those previously reported^28^. In addition, it was found that PRP had ability to improve testosterone levels in diabetic rats^43^. However, the current study recorded that i-PRF treatment was the most effective in increasing testosterone, estradiol and FSH levels after T/D comparing to PRP treatment. PRP treatment was able to induce a higher level of LH than that of i-PRF.

Our results showed significant increase of Caspase-3 expression in T/D group. In addition, the expression of TNF-α and IL-1β was also elevated in T/D group. These results were in accordance with those previously reported^44^. Parallel to our results, the previous results^11^ using ELISA confirmed the high TNF-α and Caspase-3 levels in T/D group. In a previous study investigating the relation between apoptosis due to testicular torsion and active caspase-3, 8, and 9 expressions, found a significant increase in expression of all caspase types after I/R injury. They also reported morphological and biochemical alterations due to lipid peroxidation formation^45^. Other authors confirmed this finding where they stated that testicular torsion causes germ cell apoptosis and loss of spermatogenesis. It was reported that massive germ cell apoptosis occurs in a relation with increased testicular oxidative stress that happens subsequent to reperfusion, as well as a shift in mitochondrial respiratory chain activity. These changes cause loss of spermatogenesis^46^. Apoptosis of the germ cells in testicular tissue may be evaluated by levels of caspase-3 in testicular tissue^7,37^.

In the present work, the expression of Caspase-3 protein in the testicular tissue was substantially higher in the T/D group and decreased following PRP and i-PRF therapy as compared to the T/D group. Similar findings belonging the use of PRP in treatment of testicular torsion were reported previously^28^. Treatment of such condition with i-PRF was more efficient in downregulation of TNF-α and Caspase-3 gene expression after T/D injury when compared to PRP treatment as shown in the present study. It was reported that i-PRF has anti-inflammatory activity with the ability to downregulate the inflammatory markers including TNF-α and IL-1β^22,47,48^.

TNF-α and IL-1β secretion operate as pro-inflammatory cytokines, causing the synthesis of IL-6 and activating stress-related intracellular and extracellular pathways^49^. These cytokines found to be generated by both testicular cells and activated interstitial macrophages^50,51^. TNF-α, in particular, is a powerful regulator of both normal and pathological apoptosis. IL-1β production, like TNF-α, activates inflammatory pathways when it is triggered by adequate extracellular stimuli.

In T/D group, histopathological investigation revealed thickened testicular capsule, atrophied seminiferous tubules with thickening of its basal lamina, severe necrosis and loss of normal seminiferous tubules organized lining epithelium, loss of several types of spermatogenic cells especially spermatocytes II, spermatids and sperms. Also, some seminiferous tubules were completely devoid from any lining cells. In addition, sever dilatation of the intertubular blood vessels, congestion, and severe vacuolar degeneration accompanied with severe leydig cells hyperplasia were demonstrated. These investigations were going hand in hand with previous study^27^ where they reported markedly thickened testicular capsule with wide fibroses of parenchyma after long torsion/detorsion (LTD). Also, these findings were in parallelism with previously reported study^52^ who claimed wide spread denudation, desquamation and focal necrosis in the testicular seminiferous tubule epithelia of T/D group.

The group treated with PRP had a moderate therapeutic effect after T/D. They clarified intact testicular parenchyma, but with moderate histological changes as moderate spermatogenic cell necrosis, moderate congestion of the intertubular blood vessels. But, these findings were partially similar to previous study^27^ who reported improvement of testicular histoarchitecture after PRP treatment with restoration of seminiferous tubules histoarchitecture and lined with intact germ cells. The interstitial area of the testes containing normally appeared Leydig cells with large round nuclei.

From our research, this study is the first to investigate the therapeutic efficacy of i-PRF in regeneration of testicular germ cells damage after T/D. The results revealed a superior therapeutic efficacy of i-PRF than PRP after T/D the injury. Several researches have been investigated the efficacy of i-PRF in regeneration of bone and soft tissues in comparison to PRP^16,53,54^, but there was not any research about its efficacy in testicular torsion. The superior efficacy of i-PRF of regeneration might be attributed to much released growth factors from i-PRF than PRP with longer time of releasing^55^. In addition, more incorporation of leucocytes in i-PRF preparations than PRP was reported with ease, simple and rapid preparation without additives^53,56^. The use of PRP and i-PRF therapies has many advantages over the use of other products as they are autologous and non-immunologic reaction with ow cost^24,57^.

The use of PRP and i-PRF as therapeutic modalities for regeneration of testicular damage after high degree of testicular torsion at 1080° for 3 h in Wister rats through decreasing in MDA, Caspase-3, TNF-α and IL-1β and increasing in CAT, GPx and SOD with improving the histological structure of the testes. The regenerative effect of i-PRF was superior to PRP in restoration of histoarchitecture in the testis following T/D injury.

## Methods

All experiments performed and reported here were in accordance with the relevant guidelines and regulations and the ARRIVE guidelines^58^. The experiments were approved by the Animal Committee of Zagazig University (approval number ZU-IACUC/2/F/304/2023).

### Animals

Forty mature male Wister rats weighing (220-250gm) were kept for 2 weeks before surgery at 12 h light/dark cycles for acclimatization to the laboratory conditions. Food and water were provided ad libitum throughout the study. The animals were arranged in 4 groups; (1) Control group: the rats were left without any interference; (2) T/D group: the ratś testis were torsed at 1080° clockwise for 3 h then detorsed; (3) T/D + PRP group: the ratś testis were injected with 10 μl PRP 3 h after detorsion of the testis; (4) T/D + i-PRF group: the ratś testis were injected with 10 μl i-PRF 3 h after detorsion of the testis.

### Preparation of PRP

Through the peri-orbital vein of the rats in PRP group, 2 mL whole blood samples were drawn in 3.8% sodium citrate containing centrifugation tubes to prevent clotting. The blood samples centrifuged at 300× g for 15 min to separate the plasma from eryhtrocytes and lecukocytes and then the plasma was centrifuged at 650× g for 15 min to obtain PRP^27^. Activation of PRP was achieved by the addition of CaCL2 10% immediately before its injection.

### Preparation of i-PRF

Through the peri-orbital vein of the rats in i-PRF group, 4 ml of whole blood samples were withdrawn in plain centrifugation glass tubes without anti-coagulant and subjected to centrifugation at 700 rpm (60 g force) for 3 min as described previously^53^ to obtain i-PRF at the tip of the tubes ready for injection within 15 min after preparation.

### Experimental procedures

**Figure 8:**
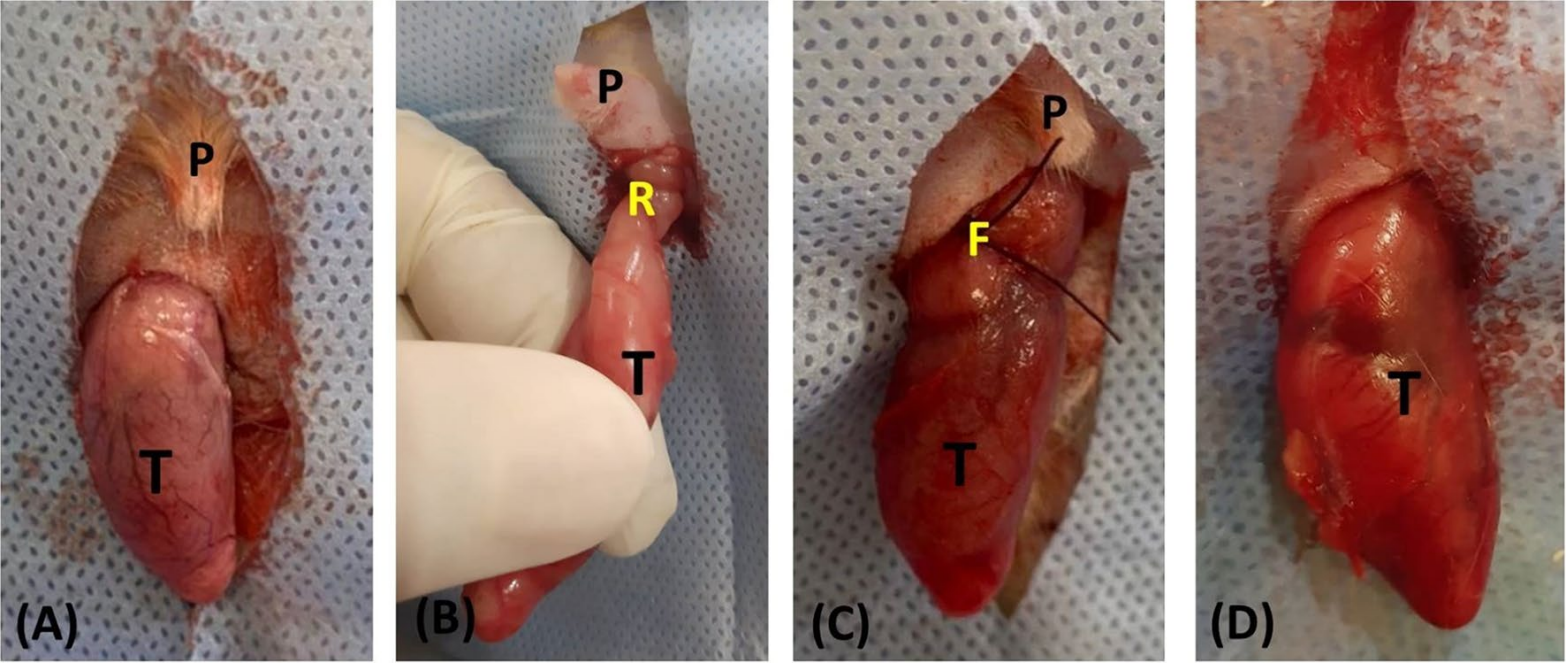
The surgical procedures used for the induction of testicular torsion. A left orchiectomy was performed via midline scrotal incision (**A**). To induce ischemia, the right testis was brought out through the scrotal incision, twisted 1080° clockwise (**B**), reinserted, and the internal spermatic fascia was fixed to the inner scrotal wall with 3/0 prolene suture (**C**); the congested testis just prior to de-torsion (**D**). (P: penis; T: testis; R: torsion with 1080°; F: fixing stitch.).

With strict asepsis, the surgical procedure of testicular torsion and detorsion was carried out as described previously^27^ under general anesthesia using intramuscular injection mixture of 50 mg/kg ketamine HCL (Ketalar®; Pfizer Egypt S.A.E. Cairo under Authority of Pfizer Inc., USA&UK.) and 5 mg/kg xylazine HCL (Xylaject; Adwia, Egypt). To avoid endocrine contribution of ipsilateral testis, left orchiectomy was performed to the rats of group 2, 3 and 4 through a median incision. The right testis was torsed at 1080° clockwise through the median scrotal incision and then detorsed after 3 h from its torsion and replaced into the scrotum (Fig. 8). Three hours after detorsion, the testis was injected with 10 μl PRP in group 3 and 10 μl i-PRF in group 4. After 30 days from surgery, the rats were sacrificed for semen evaluation, hormonal assay, gene expression of oxidative stress and histological examination.

### Semen evaluation

The sperms from the cauda epididymis of all rats of each group were collected in 2 mL 0.9% NaCl solution containing sterilized Petri dish. At 400× magnification, the sperm motility percentage was calculated according the protocol previously described^59^. The sperm concentration (cells/milliliter) was obtained using the previously described technique^60^. Following the Filler procedures, sperms abnormalities could be determined. For the determination of the head, neck/mid-piece, and tail anomalies, 500 sperm cells have been checked per rat.

### Hormonal assay

Blood samples were harvested from the sacrificed rats into clean plain tubes to obtain sera. Rat ELISA Kits from CUSABIO were used for determination of endogenic rat testosterone (Catalog Number. CSB-E05100r), luteinizing hormone (LH) (Catalog Number. CSB-E12654r), follicular stimulating hormone (FSH) (Catalog Number. CSB-E06869r), and Estradiol concentrations.

### Assay of oxidants and anti-oxidants in testicular tissue

Testicular tissues were used for preparation of testicular tissue homogenate. For gene expression analysis (RT-qPCR), rat testis were collected from all groups and immediately frozen in liquid nitrogen then stored at − 80 °C. The levels of MDA, GPx, SOD, CAT in testicular tissue were determined using a testing kit (Bio-diagnostic Giza, Egypt) as directed by the manufacturer with (CAT. No. CA 25 17) for CAT, (CAT. No. SD 25 21) for SOD, (CAT. No. GP 2524) for GPx, and (CAT. No. MD 25 29) for MDA.

### Real-time quantitative PCR (RT-qPCR) analysis

From the frozen testis at − 80 °C, total RNA using the Qiagen RNeasy® Mini kit was isolated. Quantity and quality of RNA was determined by Spectrostar Nano drop. Following manufacturer's protocol for High Capacity cDNA Reverse Transcription Kits, single-stranded cDNA from 1000 ng of total RNA was made (Applied Bio systems). The temperatures were 25 °C for 10 min, followed by 37 °C for 120 min, and then 85 °C for 5 min while cycling. Forward and reverse primers used to investigate genes by real-time PCR were listed in Table 2. The comparative CT method was used to calculate changes in gene expression provided by real-time PCR instrumentation to a reference (housekeeping) gene (β-actin)^61^.

**Table 2:** Forward and reverse primers sequences used for real-time PCR.

Gene	Primer sequence	
IL-1β	Forward	5′-CATCTTTGAAGAAGAGCCCG-3′
	Reverse	5′-AACTATGTCCCGACCATTGC-3′
TNF-α	Forward	5′-AAATGGGCTCCCTCTCATCAGTTC-3′
	Reverse	5′-TCCGCTTGGTGGTTTGCTACGAC-3′
Caspase-3	Forward	5′-GTGGAACTGACGATGATATGGC-3′
	Reverse	5′-CGCAAAGTGACTGGATGAACC-3′
β-actin	Forward	5′-AGAAGAGCTATGAGCTGCCTGACG-3′
	Reverse	5′-CTTCTGCATCCTGTCAGCGATGC-3′

### Histological and histochemical processing

Immediately after sacrificing, testicular samples were fixed in 10% neutral buffered formalin for 24 h for histological examination, then dehydrated in ascending grades of ethyl alcohol, cleared in benzene and imbedded in paraffin blocks. 5 µ sections were stained with Harris's Hematoxylin and Eosin (H&E) for routine histological studies, Blue Masson’s Trichrome for collagen fibers demonstration, and Periodic acid–Schiff (PAS) for detection of glycogen and neutral muco-polysaccharides. Following standard protocols described previously^62^ histological and histochemical stains were performed.

### Statistical analysis

The SPSS software (Version 17, Chicago, IL) was used for statistical analysis. With mean ± standard deviation (SD) the data were expressed and analyzed using the one-way analysis of variance (ANOVA) and Tukey Post-Hoc Test. the Kruskal–Wallis non-parametric statistical test is performed if the data is not normally distributed, followed by the Mann–Whitney. The results of *p* < 0.05 were considered significant.

## Data Availability

The data that support the findings of this study are available from the corresponding author upon reasonable request.
